# A New Tool for Complement Research: *In vitro* Reconstituted Human Classical Complement Pathway

**DOI:** 10.3389/fimmu.2018.02770

**Published:** 2018-12-04

**Authors:** Michele Mutti, Katharina Ramoni, Gábor Nagy, Eszter Nagy, Valéria Szijártó

**Affiliations:** Arsanis Biosciences, Vienna, Austria

**Keywords:** C1-INH, bactericidal activity, monoclonal Ab, classical pathway, alternative pathway, *E. coli* ST131, MG1655, complement system

## Abstract

The complement, as part of the innate immune system, represents the first line of defense against Gram-negative bacteria invading the bloodstream. The complement system is a zymogen cascade that ultimately assemble into the so-called membrane attack complex (MAC), which lyses Gram-negative bacteria upon insertion into the outer membrane. Traditionally, serum has been used as complement source, for example to study the bactericidal activity of monoclonal antibodies or antibodies raised upon vaccination. Due to the significant donor to donor variability, as well as susceptibility of complement factors to handling and storage conditions, assay reproducibility using human serum is low. Moreover, the presence of pre-existing antibodies and antimicrobial compounds are confounding factors. To remove antibodies from human serum, we applied κ/λ-light chain specific affinity chromatography, however the method severely reduced the complement activity due to the depletion of complement components. Therefore, we attempted to reconstitute human complement—namely the alternative (rAP) and the classical (rCP) pathways—from purified complement factors. We found that adding C1-inhibitor to the mixture was essential to maintain a stable and functional C1 and thus to generate an active rCP. We further confirmed the functionality of the rCP by testing the complement-dependent bactericidal activity of a human monoclonal antibody, A1124 against an *E. coli* clinical isolate belonging to the ST131 clonal complex, and that of a polyclonal IVIg against a laboratory *E. coli* strain (MG1655) not expressing LPS O-antigen and capsule. Although the alternative pathway did not have any bactericidal activity by itself, it enhanced MAC deposition induced by rCP and increased the overall bactericidal activity against the ST131 *E. coli* strain. In conclusion, we report for the first time the successful *in vitro* reconstitution of the classical pathway of the human complement to establish a serum-free, complement dependent bactericidal assay. This system offers high level of standardization and could support the study of the complement in different research fields.

## Introduction

As part of the innate immunity, the complement system is one of the first lines of defense against Gram-negative pathogens. Besides its direct bactericidal activity, the activation of the complement system stimulates phagocytosis and triggers pro-inflammatory signaling. The three different pathways—alternative, classical and lectin—converge into the terminal pathway (TP) that leads to the assembly of the C5b-9 complex, also called Membrane Attack Complex (MAC). The alternative pathway (AP) is spontaneously activated on the pathogen surface. The classical (CP) and lectin pathways (LP), are induced by an initial receptor-ligand recognition on the target surface. In case of the CP, the antigen-antibody complex (or immune complex, IC) activates the complement cascade by binding to C1, a complex formed by C1q, C1r, and C1s. In the LP, various lectins recognize specific sugar structures on the microbial surface and activate the Mannose Binding Protein-Associated Serine Proteases (MASPs) (1). Both CP and LP are negatively regulated by the C1 inhibitor (C1-INH), which sequesters and inhibits C1r, C1s, and MASPs (2). In the absence of C1-INH, C1 undergoes spontaneous activation (3, 4). Cleavage and activation of the various complement factors leads to the formation of the C5 convertase cleaving C5 to C5a and C5b, initiating the terminal pathway. C5b, attached to the target surface, associates with C6, C7, and C8 and induces the polymerization of C9 monomers, forming the MAC. MAC is a pore forming complex able to insert into lipid membranes, including the outer membrane of Gram-negative pathogens to induce membrane damage and cell lysis.

The different complement pathways interact with each other establishing a complex network in the serum (1, 5, 6). This complexity is further increased by the presence of additional serum bactericidal factors that may act in concert with the complement, or work independently against the invading pathogens (7–9). Therefore, it is difficult to dissect the contribution of the different complement pathways to the bactericidal action of the serum. It is especially relevant for studies using human serum as complement source due to the heterogeneity of the pre-existing antibody repertoires against human pathogens (10) and complement activity (11). While previous studies reported the successful reconstitution *in vitro* of the alternative pathway from individual components (12, 13), up to our knowledge, there have been no reports of a functionally active reconstituted classical pathway, particularly not to study its bactericidal activity.

We aimed to establish an *in vitro* model system with purified human complement components that allows studying complement without confounding factors.

## Methods and Materials

### Bacterial Strains and Media

*E. coli* strains 81009 (14, 15) and MG1655 (16) were grown in LB or on Trypticase soy agar (TSA) plates (bioMérieux). To prepare overnight cultures, a single colony was inoculated in LB and incubated at 37 °C with agitation at 200 RPM overnight. Mid-log-phase bacterial cultures were obtained by diluting the overnight cultures 1:100 in LB and incubating at 37°C at 200 RPM until the cultures reached an OD_600_ of ~0.5.

### Complement Factors, Sera, and Reagents

Rabbit erythrocytes, Gelatin Veronal Buffer with or without EDTA, as well as the complement factors C3 (A113c), H (A137), I (A138), B (A135), P (A139), C5 (A120), C6 (A123), C7 (A124), C8 (A125), C9 (A126), C1 (A098), C2 (A112), C4 (A105c), and C1-INH (A140) were purchased from CompTech. Dullbecco's phosphate-buffer saline (DPBS) without (14190144) or with calcium and magnesium (14040133) was obtained from ThermoFisher Scientific. Human serum albumin (HSA) (Albiomin, 200 g/L) was from Biotest. The monoclonal antibody A1124, a humanized IgG1 targeting the LPS O25b-antigen of *E. coli* ST131-H30 strains, as well as an isotype-matched control mAb were expressed in CHO cells as described by Guachalla et al. (17). Blood was taken from healthy volunteers into Vacutainer® clot activator tube (367896, Becton Dickinson and Company), and the off-the-clot normal human serum (NHS) was separated by centrifugation and aliquots were stored at −80°C until use. ClairYG® (50mg/mL) was obtained from LFB-Biomedicaments.

### Determination of Complement Activity

The complement activity was measured by commercial ELISA-based kits from Eurodiagnostica detecting C5b-9 deposition specific to classical (Wieslab COMPL CP310 RUO), alternative (Wieslab COMPL AP330 RUO) and MBP (Wieslab COMPL MP320 RUO) pathways according to manufacturer's instructions. Hemolytic activity of the complement was determined using rabbit erythrocytes according to the manufacturer's instructions using NHS as positive control.

### Removal of Antibodies From Human Serum

To remove antibodies from NHS, κ/λ-light chain specific affinity chromatography was employed. The matrix was prepared by mixing three parts of κ-light chain specific beads with one part of λ-light chain specific beads (083310 and 084910, respectively, Life Technology). To avoid clogging of the resin, NHS was spin-filtered before incubating with double volume of resin at 4°C for 10min. The supernatant was separated from the matrix by centrifugation (Ig depleted serum, Ig-dep). As control, equivalent volume of serum was treated identically but without the chromatography matrix (mock). Both Ig-depleted and mock sera were concentrated with Vivaspin 500 (10,000 MWCO PES, Sartorius). The dilution factor was determined based on absorbance measurement at 405, 490, and 650 nm, compared to that of untreated serum. To remove beads, fibers and precipitates, the samples were spin-filtered. Bound antibodies were recovered by washing the resin with DPBS with Ca/Mg, eluting with 0.1M glycine pH 2.0 buffer and neutralizing with 1M Tris pH 8.0. This immunoglobulin fraction was concentrated with Vivaspin 20 (10,000 MWCO PES, Sartorius) followed by Vivaspin 500 and finally spin-filtered. In all cases Ultrafree™-MC Centrifugal Filter Devices 0.22μm (EMD Millipore) was employed for spin-filtration. Samples were stored at −80°C.

### Preparation of Fab Fragment of Human IVIg

Fab fragments of human IVIg (ClairYg®) were prepared by digestion with agarose-immobilized papain (20341, ThermoFisher) according to the manufacturer's instructions. DPBS with 10mM EDTA pH 7.0 was used as buffer. The uncleaved Igs and Fc fragments were removed by using MabSelect Sure® (17543801, GE Healthcare) according to the manufacturer's instructions. The flow-through, containing the Fabs, was buffer exchanged in DPBS, concentrated (Vivaspin 500, Sartorius) and spin filtered (Ultrafree™-MC Centrifugal Filter Devices 0.22μm; EMD Millipore) to ensure sterility. An untreated aliquot of ClairYg® was buffer exchanged and treated the same way to be used as control. The concertation of both Fab and full IgG in DPBS was determined by bicinchoninic acid (BCA) assay (23225, ThermoFisher).

### Surface Staining of *E. coli*

To detect surface binding antibodies, 10^8^ CFU/ml bacteria were incubated with various concentration of ClairYg® or its Fab fragments, followed by incubation with secondary detection reagent Alexa Fluor 488 goat anti-human IgG Fcγ or Alexa Fluor 488 goat anti-human Fab_2_ (109-546-170 and 109-546-097 respectively, Jackson Immuno) and stained with 5μM SYTO 62 nucleic acid stain (S11344, ThermoFisher). Samples were measured in a CytoFLEX flow cytometer (Beckman Coulter) and data were analyzed using the FCS Express Flow 5 (*De Novo* Software).

### Immunoblotting

Samples diluted 1:400 in Laemmli buffer were separated on Mini-PROTEAN TGX 4-20% gels (4561096 or 4561094, Bio Rad) and blotted onto 0.22μm nitrocellulose membrane using the high MW program of the TransBlot Turbo system (Bio Rad). The membrane was blocked in 5 % skim milk in Tris Buffered Saline (TBS, Fischer scientific), incubated with 1:50,000 diluted horseradish peroxidase (HRP) conjugated goat anti-human IgG (H+L) (109-035-088, Jackson ImmunoResearch) and 1:50,000 HRP conjugated goat anti-human albumin (PA1-28334, Pierce). To detect C4 cleavage, the membrane was incubated with goat anti-human C4 serum (1:2,000, A205, CompTech) as primary antibody and with donkey IgG-HRP anti-goat-IgG (1:40,000, 6420-05, Southern Biotech) as secondary antibody. Blots were developed with Amersham ECL Prime solution reagent (GE Healthcare).

### Determination of C1q and MBL2 Levels in Human Sera

Concentrations of C1q and MBL2 were determined by ELISA using a commercial kit according to the manufacturer's instructions (HK356 and HK323, respectively, HycultBiotech). The serum samples were diluted 1:200 for C1q and 1:50 for MBL2 determination. The Ig depleted sera and the eluates were diluted according to the estimated protein concentrations.

### Preparation of C1/C1-INH Mixture

C1-INH was concentrated to about 2 mg/mL (Vivaspin 500, Sartorius), mixed with about 0.2–0.4 mg/mL C1 and 25 mg/mL HSA, and incubated for 10min on ice. This mixture or C1 alone was dialyzed twice against DPBS with Ca/Mg at 4°C for 3 h followed by concentration to ~1.3 mg/mL of C1 (Vivaspin 500, Sartorius). In the C1/C1-INH mixture, the C1-INH to C1 ratio was 1.8 times the physiological ratio. The samples were spin filtered to ensure sterility (Ultrafree™-MC Centrifugal Filter Devices 0.22μm; EMD Millipore) and then aliquoted and stored at −80°C until use. At least of three different batches of C1/C1-INH were prepared and used.

### Complementation Assay With C1q-Depleted Serum

C1 alone or premixed with C1-INH was incubated on ice or at 37°C for 60min, and added to C1q-depleted serum (A300, CompTech). The activity of the classical pathway was measured with the ELISA-based kit described above.

### C4 Cleavage Assay

Heat-aggregated IgG (HAGG) was generated by incubating ClairYg® in DPBS with Ca/Mg at 63°C for 30min as described previously (18). C4 (200μg/mL), HSA (20mg/mL) and HAGG (750μg/mL) were mixed on ice (final concentration indicated). C1 (67.5 μg/mL) combined with C1-INH at various final concentrations ranging between 68.5 and 685μg/mL was added to the mixture. The concentration of Ca and Mg were kept constant at 0.45mM and 0.25mM, respectively. After 5min on ice, the reaction was started by incubation at 37°C for 5min. The reaction was stopped by boiling in reducing Laemmli buffer (1610737, BIORAD for 5 min. C4 cleavage was detected by immunoblotting as described above.

### Reconstitution of the Human Complement

The alternative pathway (rAP) was reconstituted as reported by Schreiber et al. (12, 13). Briefly, C3, factor H and factor I were incubated for 30min at 37°C. After cooling the mixture on ice, factor B, factor P, and the terminal pathway components C5, C6, C7, C8, and C9 were added. For the reconstitution of the rCP, C3 was incubated for 30min at 37°C (analogous to treatment in rAP), cooled on ice, then TP components, C2, C4, and C1/C1-INH mixture were added, and then the solution was supplemented with DPBS and HSA. For the combination of the alternative and classical pathways (rAP+rCP), the alternative pathway was reconstituted as above, then TP components, C2, C4, and C1/C1-INH mixture were added, and finally the mixture was supplemented with DPBS and HSA. In all experiments the final concentration of Ca, Mg and HSA was 0.17 mM, 0.09 mM, and 25 mg/mL respectively. The proteins were added in the order described above. The final concentration of each factor matched the physiological concentration of 50% diluted human serum (3, 12): C3 at 600μg/mL, C5 at 36μg/mL, C6 at 32 μg/mL, C7 at 27μg/mL, C8 at 27 μg/mL, C9 at 29.5 μg/mL, factor B at 100 μg/mL, factor D at 1μg/mL, factor H at 235μg/mL, factor I at 17μg/mL, factor P at 10μg/mL, C2 at 12.5μg/mL, C4 at 200μg/mL, C1 at 67.5μg/mL and C1-INH at 120μg/mL.

### Activity of rCP With or Without C1-INH

When the rCP with or without C1-INH was tested, the preparations were treated as follows. Two mixtures of rCP with or without C1-INH were prepared simultaneously as described above, on ice and aliquoted into 5 tubes. One aliquot was snap frozen in liquid nitrogen and stored at −80°C. The remaining four aliquots were incubated at 37°C or on ice for 10min or 60min and then snap frozen. For measuring complement activity, the aliquots were thawed on ice at once and the assay was started immediately.

### Bactericidal Assay

Survival of bacteria was assayed as described previously (19). Briefly, bacteria at about ~5 × 10^4^ CFU/mL (collected from mid-log phase culture) were added to the reconstituted complement and incubated at 37°C with agitation. After 3 h, surviving bacterial count was enumerated by plating. In case of *E. coli* strain MG1655, the final concentration of each factor matched the physiological concentration in 25% diluted human serum, while in assays with the *E. coli* strain 81009, the factors were set to 50% of the physiological concentration.

### Stability of rCP

rCP mixture was divided into four aliquots; one was immediately frozen at −80°C (untreated control), one underwent 3 cycles of freeze and thaw, one was stored at 4°C for 48 h, and one was incubated at 37°C for 24 h before freezing at −80°C. On the day of the testing, the samples were thawed at once and their complement activity was compared with the ELISA based kit as described above.

### Statistical Analysis

Data were analyzed with paired two-tailed *t*-test using GraphPad Prism version 6. Difference was considered statistically significant if *P* < 0.05.

## Results

### Removal of Antibodies From Human Serum Impairs the Complement

**Figure 1:**
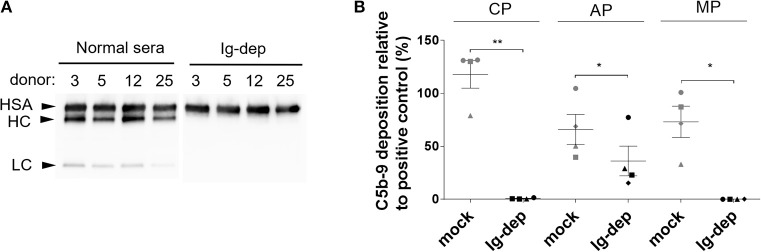
Depletion of Ig from normal human serum. **(A)** IgG (Fcγ-heavy chain, HC and light chain, LC) was detected by immunoblotting in normal human serum (NHS) and in the corresponding sera depleted with light-chain specific resin (Ig-dep). Albumin (HSA) was used as loading control and detected with a specific antibody. **(B)** Classical (CP), alternative (AP), mannose-binding lectin (MP) pathway activity was measured by ELISA in sera depleted with light chain specific resin (Ig-dep), or in mock treated (mock) samples and compared to a positive control provided in the kit. Graph shows mean values obtained with sera from 4 donors ±SEM. C5b-9 deposition in Ig-dep was compared to the mock treated sample by two-tailed paired *t*-test (*P < 0.05; **P < 0.01). The circle, square, triangle and diamond represent donor #3, #5, #12, and #25, respectively.

First, we tested the possibility of removing antibodies from normal human serum (NHS) to generate a complement source devoid of pre-existing antibodies for studying complement-mediated bactericidal activity. We employed affinity chromatography with Ig light chain (κ and λ) specific resin to remove all isotypes of antibodies. Based on immunoblot analysis, no IgGs could be detected in the depleted sera (Figure 1A). Next, we determined whether Ig depletion affected the complement activity of the human serum samples. The four NHS tested had comparable classical and alternative pathway activities prior to depletion, however, all had reduced or no activity after incubation with the resin (Figure 1B). We detected both MBL2 and C1q in the elution fractions together with the antibodies (Supplementary Figure 1).

Given that depletion of antibodies from NHS was not possible without compromising the complement activity, we next attempted to reconstitute the classical and the alternative complement pathways *in vitro*.

### *In vitro* Reconstitution of the Alternative Complement Pathway

**Figure 2:**
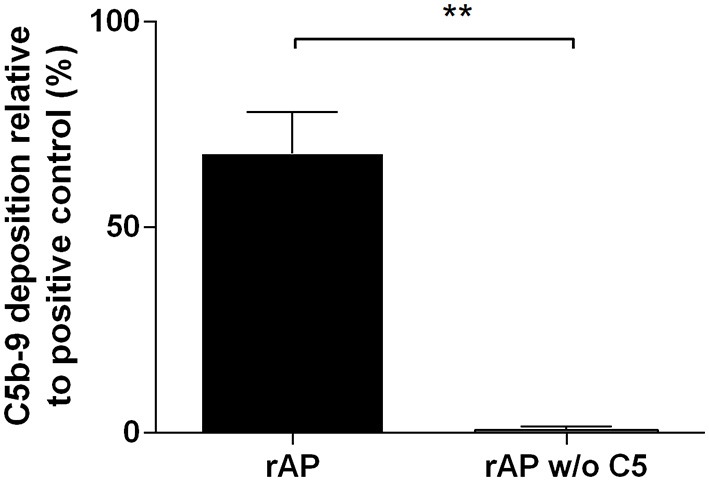
Activity of reconstituted alternative pathway. The activity of the reconstituted alternative pathway with all factors (rAP) or without C5 (rAP w/o C5) was measured by ELISA. The bars show mean ± SEM of 3 independent experiments performed with duplicates. Results were compared with paired *t*-test (**P < 0.01).

First, we wanted to recapitulate the results of Schreiber et al. (12) reported four decades ago demonstrating the functional activity of reconstituted alternative pathway (rAP). We combined purified complement components in the following order: C3, factor H and factor I incubated at 37°C, then adding factor B, factor P, and the terminal pathway components C5, C6, C7, C8, and C9. As a measure of complement activity, we detected C5b-9 assembly by ELISA (Figure 2) or by the rabbit erythrocyte lysis assay (Supplementary Figure 2). The activity of the rAP measured by ELISA was in the range of the AP activity of untreated NHS samples and the rabbit RBC lytic activity of rAP was also comparable to that of NHS (Supplementary Figure 2). The activity in both assays was specific, as it was completely lost upon the exclusion of the C5 component from to the rAP mixture.

### C1-INH Is Required to Preserve the Functionality of the Classical Pathway

**Figure 3:**
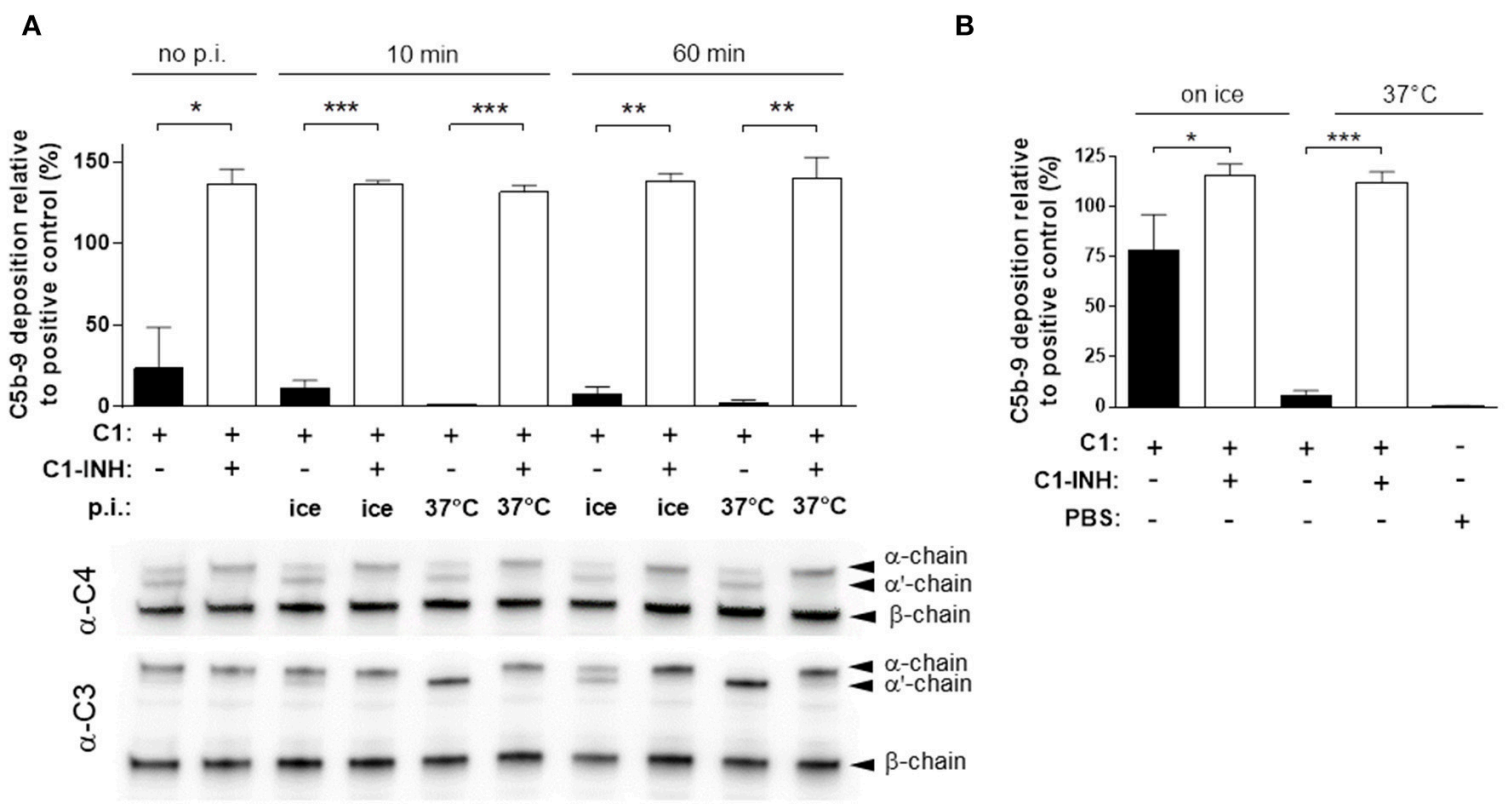
Preserving C1 activity by C1-INH. **(A)** The activity of the reconstituted classical pathway (rCP) was measured by ELISA. rCP with or without C1-INH was pre-incubated (p.i.) for 10 min or 60 min, at 37°C or on ice, before testing for C5b-9 deposition. Graphs show mean ± SEM from 3 experiments, compared with two-tailed paired *t*-test (*P < 0.05; **P < 0.01; ***P < 0.001). The same samples were assayed for C4 and C3 cleavage by immunoblotting that is indicated by the appearance of the α'-chain. **(B)** C1 incubated for 1 h at 37°C or on ice was added to C1q-depleted serum. The classical pathway activity was measured by ELISA detecting C5b-9 deposition. The results are expressed as % of signal compared to the positive control. Graph shows mean ± SEM from 4 independent experiments compared with two-tailed paired *t*-test (*P < 0.05; ***P < 0.001).

Analogous to the generation of rAP, we attempted to reconstitute the classical pathway (rCP) by combining all factors from C1 to C9. First, C3 was incubated at 37°C, and cooled on ice before the terminal pathway components C5, 6, 7, 8 9, then C2, C4, and finally C1 were added at concentrations equivalent to those in 50% human serum. To test the stability of the reconstituted classical pathway, the mixture was preincubated for up to 60min at 37°C, before being assayed for the C5b-9 deposition. To resemble the physiologic activation, we measured the activity of the classical pathway with an ELISA kit, with wells coated with IgM, mimicking the immunocomplexes. Surprisingly, we detected only a very low complement activity (approximately 20% that of human serum) that was even further decreased when the mixture was incubated on ice or was completely lost after incubation at 37°C (Figure 3A).

Since the rAP, that shares many components with the CP, was functionally active and stable, we focused our investigation on the components specific for the classical pathway, particularly C1. To assess the activity of C1, we employed a C1q depleted (C1q-dep) serum preparation that lacked any CP activity and supplemented this with C1. We found that C1 was able to rescue the classical pathway activity of the C1q depleted serum, but with decreased efficiency after pre-incubation of C1 at 37°C (Figure 3B). These data suggested that the purified C1 was functional, but it lost its ability to induce the C5b-9 deposition upon incubation at 37°C.

We speculated that the loss of C1 activity was due to the spontaneous activation of C1 itself. Therefore, we assessed whether the C1 inhibitor (C1-INH), a serine protease inhibitor naturally present in human serum, would be able to stabilize C1. Indeed, we observed that the supplementation of C1q-depleted serum with C1 pre-incubated at 37°C in the presence of C1-INH, fully restored the CP activity (Figure 3B). Hence, we added C1-INH to the rCP, and the experiment was repeated as described above. When adding C1-INH to the rCP, we observed a full classical pathway activity, in all the conditions tested (Figure 3A).

To monitor the C1 activity at the molecular level during the incubation of rCP, we detected C4 and C3 cleavage products by immunoblotting. We observed the proteolytic conversion of both C4 and C3 α chains to α'-chain in the absence, but not in the presence of C1-INH (Figure 3A). These data corroborated that spontaneous activation of C1 led to activation of the CP, which ultimately consumed the native complement factors in the mixture. On the other hand, in presence of C1-INH we did not observe any cleavage of C4 and C3, even after incubation for 1 h at 37°C (Figure 3A).

To examine whether the C1-INH concentration used in the assay still allows the specific activation of C1 initiated by immune complexes (IC), we monitored C4 cleavage triggered by heat-aggregated Ig. We found that even large excess of C1-INH (up to 10-fold the physiological C1-INH/C1 ratio) did not impair the specific activation of C1 (Supplementary Figure 3).

We also tested the stability of rCP upon freeze-thaw cycles or incubation at 37°C or at 4°C. As shown in Supplementary Figure 4, the rCP is resistant to three cycles of freeze and thaw, and stable at 4°C for 48 h, but incubation at 37°C for 24 h significantly impairs the complement activity.

### rCP, Alone or in Combination With rAP, Is Functional and Induces a Strong Bactericidal Action

**Figure 4:**
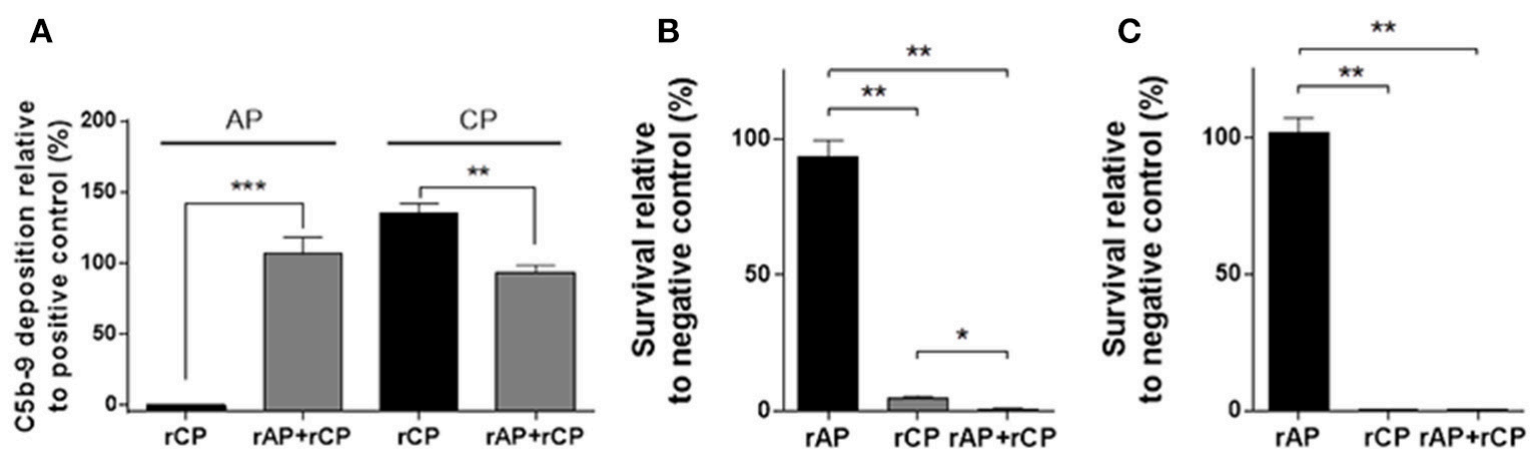
Complement and bactericidal activity of *in vitro* reconstituted complement pathways**. (A)** ELISA detecting the alternative (AP) or classical (CP) pathway-dependent C5b-9 deposition by the *in vitro* reconstituted alternative (rAP) and classical pathway (rCP), alone or in combination (rAP+rCP). The results are expressed relative to positive control. Graphs show mean values ±SEM of 6 independent experiments analyzed with two-tailed paired *t*-test (**P < 0.01; ***P < 0.001). **(B)** Survival of the *E. coli* strain 81009 in the presence of the reconstituted complement pathways with mAb A1124 after 180min incubation. The survival is expressed relative to the survival with an unrelated control mAb. **(C)** Survival of the *E. coli* strain MG1655 in the presence of the reconstituted complement pathways with ClairYg® after 180 min incubation. The survival is expressed relative to the survival in presence of ClairYg®-derived Fab. Graphs **(B,C)** show mean ± SEM of 3 and 2 independent experiments, respectively, analyzed with two-tailed paired *t*-test (*P < 0.05; **P < 0.01).

Next, we reconstituted the classical complement pathway in combination with rAP. Both rAP and rCP were functionally active when combined (rAP+rCP) (Figure 4A). However, when measuring the classical pathway activity in the combined system, it was lower than in the rCP alone (Figure 4A). These results suggested that components present in the rAP (partially) inhibit the classical pathway activity of rCP.

As proof of practical application, we tested the bactericidal activity of the rAP, rCP or their combination (rAP+rCP) against two different *Escherichia coli* strains in the presence of specific antibodies.

ST131 strain 81009 expressing the O25b LPS O-antigen and mAb A1124 specific for O25b (17) were co-incubated with the reconstituted complement. In presence of the rCP, we observed significant antibody-dependent bactericidal activity, while rAP alone did not support bacterial killing (Figure 4B). Interestingly, the mixture of rCP and rAP displayed enhanced bactericidal effect (Figure 4B).

Another *E. coli*, the K-12 strain MG1655, not expressing LPS O-antigen (rough), was incubated with a human IVIg preparation (ClairYg®) that was shown to contain antibodies binding to the surface of this strain (Supplementary Figure 5). In the presence of IVIg (500 μg/ml), the MG1655 strain was completely killed in rCP or rAP+rCP, while it survived in the rAP (Figure 4C). MG1655 also survived and even grew in rCP and rAP+rCP when incubated with ClairYg®-derived Fab, corroborating that the bactericidal activity was not intrinsic to the rCP.

Low amounts of human IgG contamination was detected in all three mixtures of reconstituted complement (Supplementary Figure 6). Contaminants were—at least partially—introduced with the human albumin. Despite the presence of contaminating antibodies, we did not detect any specific, surface binding antibodies against the two *E. coli* strains (data not shown).

## Discussion

Serum bactericidal assay (SBA) is the gold standard *in vitro* assay to assess complement-dependent bactericidal activity of antibodies raised upon active immunization (10, 20) or applied as passive immunization (17). In SBA, human or rabbit serum is used as complement source and target bacteria are incubated in presence of specific antibodies or immune serum. In such assays, bacterial survival is compared to those with a negative control antibody or non-immune serum as well as an inactivated complement source. Most often rabbit serum is preferred over human serum, because baby rabbit sera lack pre-existing antibodies (10, 20). Previous reports showed the possibility of depleting human serum from pre-existing antibodies by using Protein G affinity purification, with the drawback that C1q and C5 were significantly depleted during the process (21). Moreover, the method did not deplete IgM or IgA, two isotypes also able to activate the complement system (1, 22).

In this study, we aimed to establish a reproducible method with a complement source free of antibodies. First, we depleted human serum from all isotypes of immunoglobulins with a light chain specific resin. While we were able to remove the majority of IgGs, this treatment abolished activity of the classical and lectin pathways due to the non-specific depletion of C1q and MBL2, significantly impaired activity of the alternative pathway, and potentially depleted other complement factors by the resin.

In addition to antibodies, non-complement serum factors can also significantly contribute to the killing of bacteria in human serum (7, 8, 23). Therefore, we decided to test a bottom-up approach and reconstituted the complement system from its individual components. While reconstituted cytolytic alternative pathway was reported by two papers of Schreiber et al. ((12), (13)), up to our knowledge there are no reports of successful reconstitution of the complete classical pathway from C1 to C9, despite the significant amount of work put into reconstituting parts of it (24–31). We aimed to establish a method to reconstitute the classical pathway also in combination with the alternative pathway, to be used in studying complement mediated bactericidal activity. To make it available for the broader scientific community, we used commercially available factors purified from human serum.

First, we reproduced previous work of reconstitution of the alternative pathway (12). This was primarily done to confirm that the purified proteins were functionally active. The protocol was slightly modified with the addition of human serum albumin as carrier protein. The mixture of the AP components was active based on the detection of the C5b-9 complex measured by ELISA and the lysis of rabbit erythrocytes, and the activity was found comparable to that of human serum (32).

When we mixed the factors of the classical pathway, we detected very low CP activity. Furthermore, short incubation at 37°C (10 min) completely abolished this activity.

**Figure 5:**
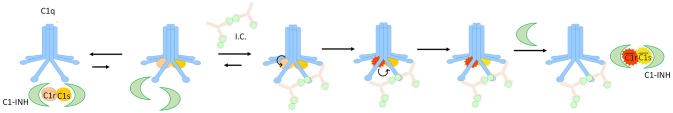
Activation of C1 in presence of C1-INH and immunocomplexes. C1-INH reversibly inhibits in fluid phase the pro-C1r and pro-C1s, when they dissociates from C1-INH they can bind C1q, but this is disfavored. In presence of immunocomplexes (I.C.), the association of C1r_2_s_2_ to C1q is favored and stable, so C1q activates C1r which in turn cleaves C1s. Afterwards, the activated C1r_2_s_2_ (indicated as $\bar{\text{C}1\text{r}}$ and $\bar{\text{C}1\text{s}}$) is then displaced from C1q and irreversibily inhibited by C1-INH.

Apart from C1, C2 and C4, all components were already used and proved to be active in rAP, therefore we tested the activity of CP-specific factors. Importantly, the commercially available C1 is supplied with EDTA and protease inhibitors, which inhibit the activity of the complement factors, therefore, we had to remove these inhibitors by buffer exchange. This treatment could have affected activity or stability of the C1 or its components, like C1q. In line with this, C1 pre-incubated on ice was able to rescue the classical pathway in C1q-depleted serum, but not when pre-incubated at 37°C. This suggested that the buffer exchange *per se* was not responsible for the impairment of C1 activity, but rather affected its stability. Furthermore, we speculated that the lack of C5b-9 deposition in both rCP and C1q depleted serum was due to the spontaneous activation of C1r and C1s at 37°C. Indeed, in normal serum, the spontaneous activation of C1 is prevented by C1-INH that has a dual role; it inhibits irreversibly the zymogen form of C1s and C1r and in a reversible way the pro-zymogen form of C1r and C1s. In the presence of ICs, C1-INH disengages the pro-zymogens C1r and C1s, allowing their activation (4) (Figure 5). Thus, we tested whether C1-INH was able to maintain a functional C1 in an *in vitro* system as well without affecting the specific activity of C1. A previous study by Nielsen (33) showed that even large excess of C1-INH had no effect on the activation of C1 by immune complexes. However, the C1-INH preparation used in that work differed from the C1-INH available for us (34, 35). Therefore, we set up a method to detect the specific activation of C1 induced by heat-aggregated gamma globulins. We observed that even a 10-fold excess of the C1-INH did not impair the specific activation by IC.

Next, we showed that the classical pathway was fully rescued when C1q-depleted serum was supplemented with C1 co-incubated with C1-INH at 37°C. Likewise, the presence of C1-INH in rCP allowed full reconstitution of the classical pathway that was unchanged even after 1 h of incubation at 37°C. We confirmed that in the absence of C1-INH, C4 was promptly cleaved, even without specific activation. The cleavage of C4 led to the cleavage of C3 as well, but with a slower kinetics. The very low activity of the classical pathway in the rCP correlated well with the amount of native C3 in the mixture. On the other hand, when C1-INH was present, both C4 and C3 remained uncleaved (in native state). In conclusion, the very low level of C5b-9 deposition by rCP in the absence of C1-INH is due to the consumption of the downstream factors, rather than to C1 instability. An interesting indirect proof for the requirement of C1-INH in CP is the phenotype of hereditary angioedema (HAE) type I and II. In HAE low activity of C1-INH leads to the activation of the contact system (which induces edema) and the collateral spontaneous activation of C1 and consumption of C4 and C2 (36).

While rCP appeared to remain active after three freeze-thaw cycles and at 4°C for 48 h, incubation at 37°C impaired the complement activity, in agreement with the observation by Mollnes et al. (37).

When we combined alternative and classical pathways, we found both to be active alone and in combination in the ELISA-based complement activity assay. The lower CP activity of rAP+rCP compared to rCP alone could be explained by the presence of Factor H and I. Factor H acts as cofactor for factor I to cleave C3b and C4b, and it can block the induction of the downstream cascade (5). In addition, factor H alone could also displace C1 from the ligand, as shown by Kishore and Sim (38).

After confirming the activity of the reconstituted *in vitro* complement, we proved that this experimental system is suitable for studying the complement mediated bactericidal activity of monoclonal or polyclonal antibodies. Using an extra-intestinal pathogenic *E. coli* strain 81009, we found that the rCP was able to induce a strong and specific bactericidal activity in combination with the specific mAb, A1124. The bactericidal activity was further increased when alternative and classical pathways were combined. This confirms that although the alternative pathway alone does not induce bactericidal effect, it enhances the activity of the classical pathway through the amplification loop. Of note, in the ELISA based activity assay we did not observe an increase in the classical pathway activity when rCP was combined with rAP, however this was due to the use of different complement dilutions in the two assays: in the ELISA the complement mixture was diluted 1:101 (vs. the 1:2 dilution used in the bactericidal assay) and at such a high dilution the contribution of the alternative pathway could not be detected.

We found that IVIg ClairYg® contained a significant amount of antibodies against MG1655, as also reported by others (39); therefore it was used as a source of Abs to target this strain. MG1655 is a rough and non-encapsulated K-12 archetype strain, extremely sensitive to the bactericidal activity of the serum. In agreement with its sensitivity, the strain was completely killed in both rCP and rAP+rCP in the presence of ClairYg®. Despite of the low content of contaminating antibodies in the reconstituted complement pathways, we did not observe any killing when no ClairYg® was added to the mixture. In contrast with our data, the K-12 derivative strain, W1485, was previously shown to be killed by a reconstituted alternative pathway alone (12). However, the growth conditions used in the two studies were different, and previously also no carrier protein was added to the reconstituted mixture. Moreover, it cannot be excluded that the complement factors used previously differed in purity and potentially contained bactericidal proteins.

To our knowledge, this is the first report of the successful reconstitution of the classical pathway from individual components. In this model system, it is possible to strictly control the concentration of each component or to substitute a factor with a mutated version, as well as to supplement the assay with additional purified components, as demonstrated by adding a specific antibody. By adding lectins and MASPs to the C1/C1-INH mixture, we speculate that the system can be further complemented with the lectin pathway.

We propose that the method could have a broad applicability beyond the testing of complement mediated bactericidal activity of antibodies. It was proposed that new methods are needed to study the role of the complement in the activity of the anti-CD20 mAB, rituximab in non-Hodgkin lymphomas (40). As serum was reported to display great donor to donor heterogeneity in the killing of cancerous cells (41), the *in vitro* reconstituted complement may offer a simple method to be used in cancer studies. Similarly, this tool could be applied to study the role of the complement in viral pathogenesis (42), transplant rejection (43) and autoimmune diseases (44, 45).

## Author Contributions

MM performed the experiments. KR provided technical assistance. MM and VS designed the experiments. MM, VS, GN, and EN wrote the manuscript.

### Conflict of Interest Statement

MM and KR are employees of Arsanis Biosciences. KR, GN, EN, and VS hold shares in Arsanis Inc, the parent company of Arsanis Biosciences.
