# Supplementary material for: A New Tool for Complement Research: In vitro Reconstituted Human Classical Complement Pathway
Source: Front Immunol. 2018 Dec 4;9:2770. doi: 10.3389/fimmu.2018.02770 (PMC6288441; doi:10.3389/fimmu.2018.02770)
Supplement: Supplementary file 1 [file Presentation_1.PPTX]

## Slide 1
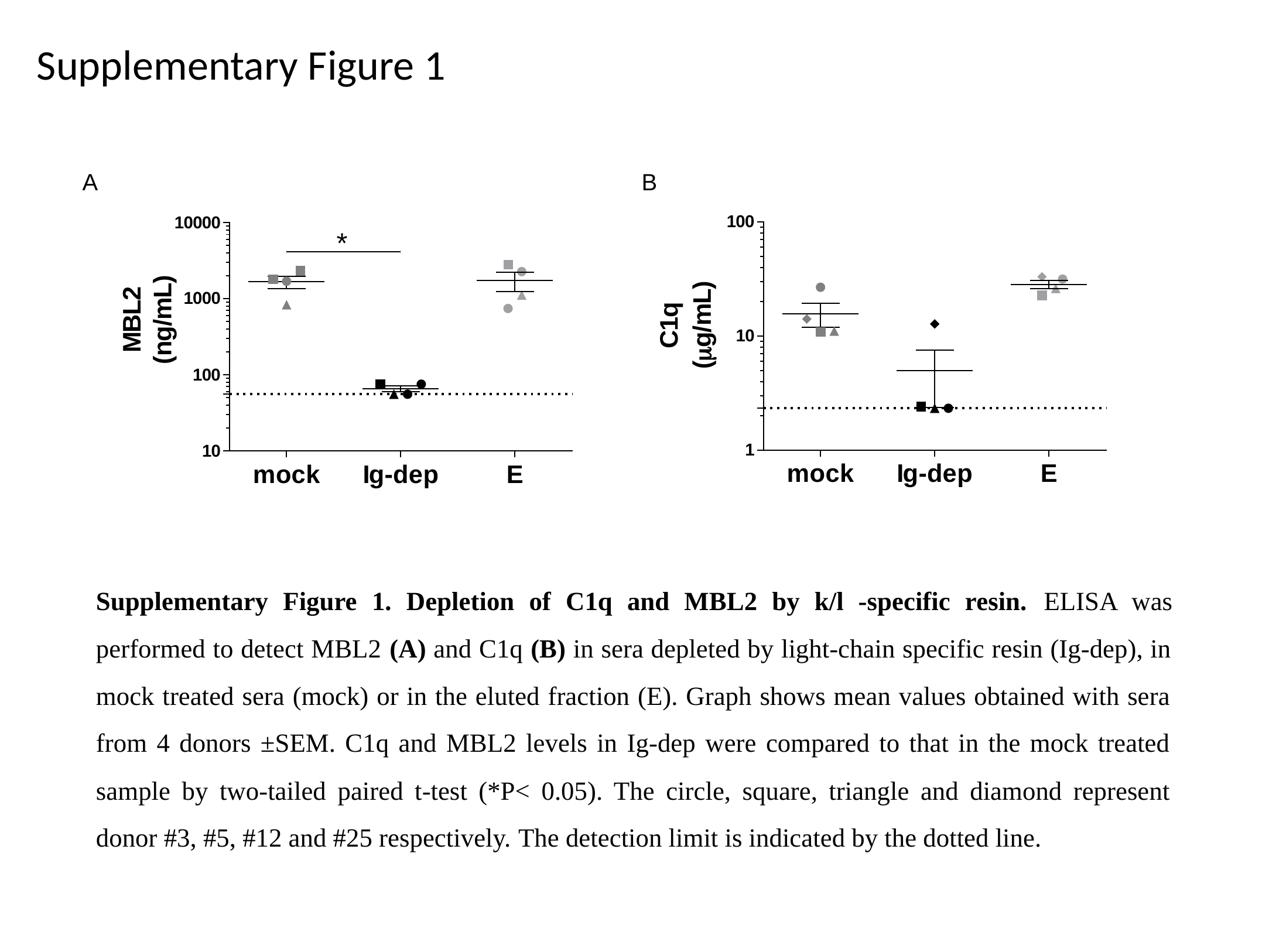

Supplementary Figure 1
A
B
Supplementary Figure 1. Depletion of C1q and MBL2 by k/l -specific resin. ELISA was performed to detect MBL2 (A) and C1q (B) in sera depleted by light-chain specific resin (Ig-dep), in mock treated sera (mock) or in the eluted fraction (E). Graph shows mean values obtained with sera from 4 donors ±SEM. C1q and MBL2 levels in Ig-dep were compared to that in the mock treated sample by two-tailed paired t-test (*P< 0.05). The circle, square, triangle and diamond represent donor #3, #5, #12 and #25 respectively. The detection limit is indicated by the dotted line.

## Slide 2
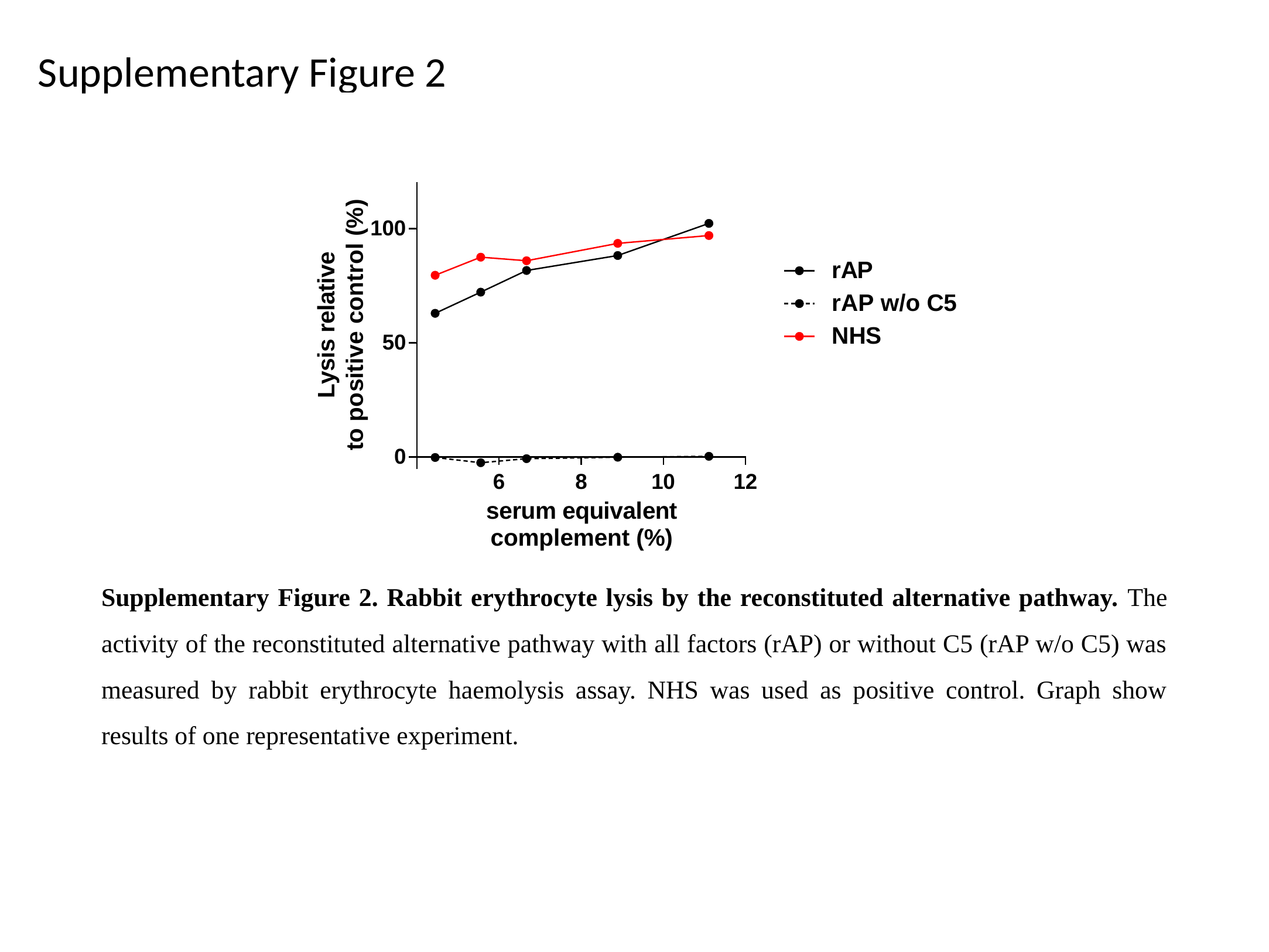

Supplementary Figure 2
Supplementary Figure 2. Rabbit erythrocyte lysis by the reconstituted alternative pathway. The activity of the reconstituted alternative pathway with all factors (rAP) or without C5 (rAP w/o C5) was measured by rabbit erythrocyte haemolysis assay. NHS was used as positive control. Graph show results of one representative experiment.

## Slide 3
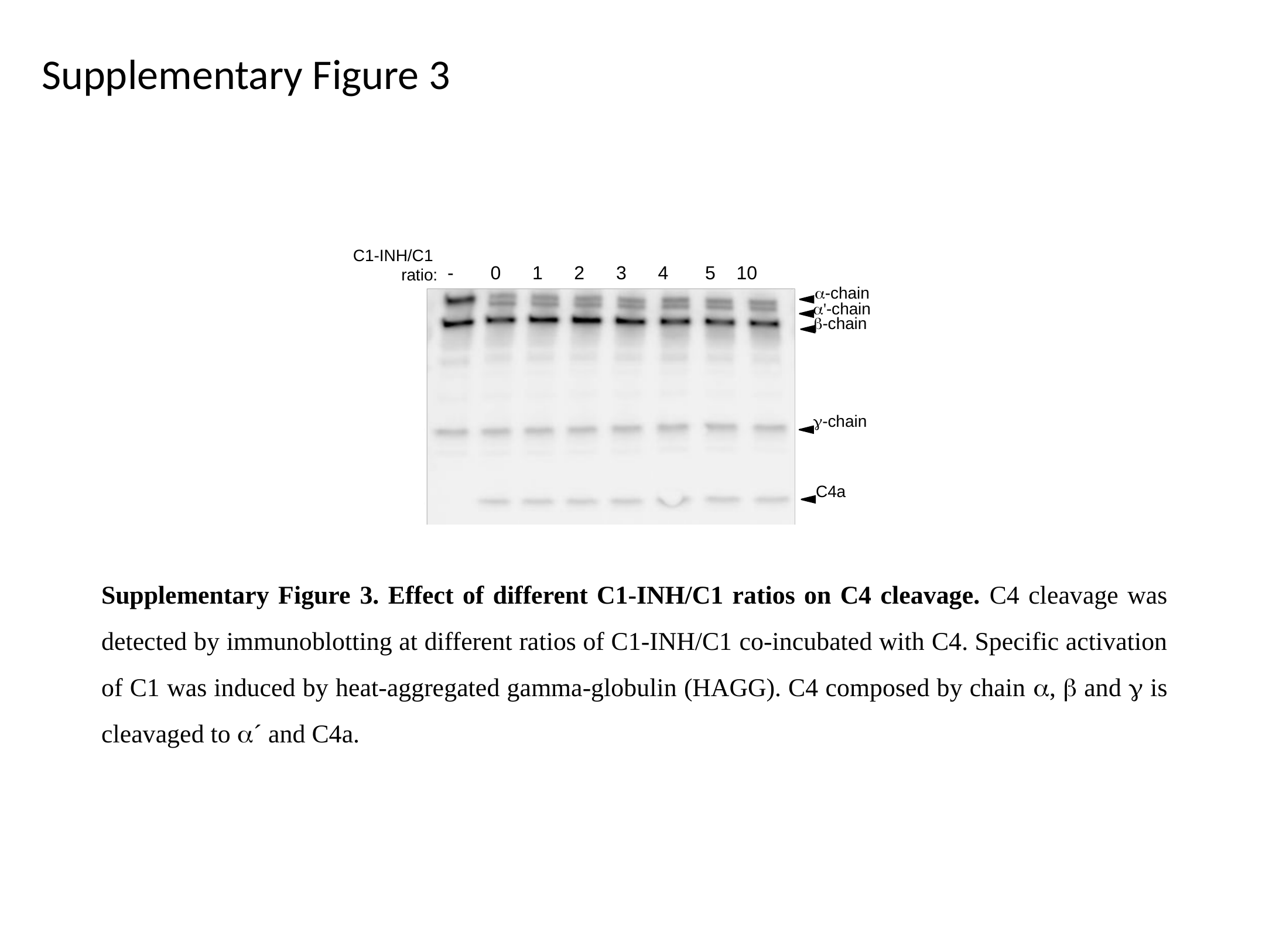

Supplementary Figure 3
C1-INH/C1
ratio:
- 0 1 2 3 4 5 10
a-chain
a'-chain
b-chain
g-chain
C4a
Supplementary Figure 3. Effect of different C1-INH/C1 ratios on C4 cleavage. C4 cleavage was detected by immunoblotting at different ratios of C1-INH/C1 co-incubated with C4. Specific activation of C1 was induced by heat-aggregated gamma-globulin (HAGG). C4 composed by chain a, b and g is cleavaged to a´ and C4a.

## Slide 4
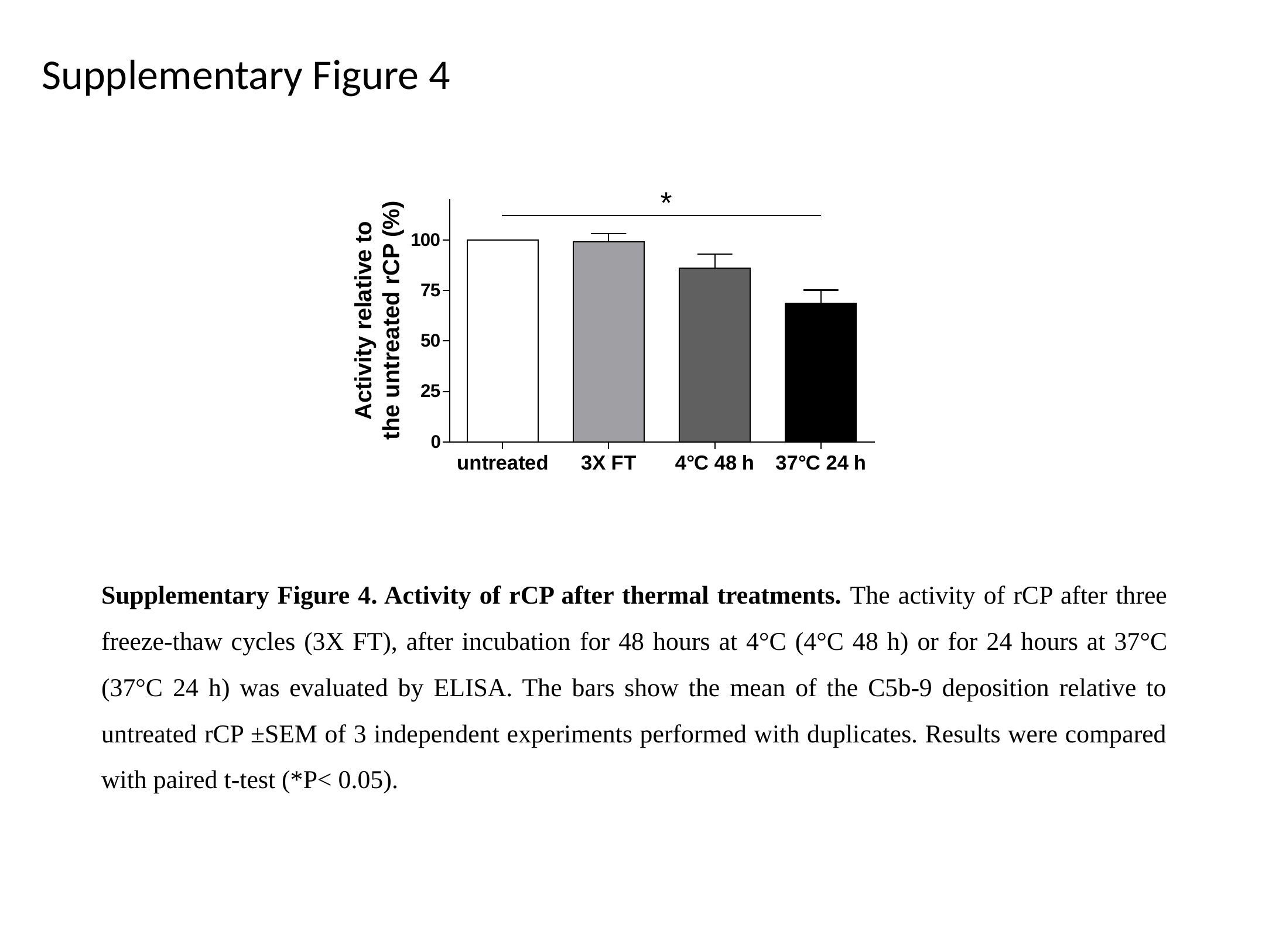

Supplementary Figure 4
Supplementary Figure 4. Activity of rCP after thermal treatments. The activity of rCP after three freeze-thaw cycles (3X FT), after incubation for 48 hours at 4°C (4°C 48 h) or for 24 hours at 37°C (37°C 24 h) was evaluated by ELISA. The bars show the mean of the C5b-9 deposition relative to untreated rCP ±SEM of 3 independent experiments performed with duplicates. Results were compared with paired t-test (*P< 0.05).

## Slide 5
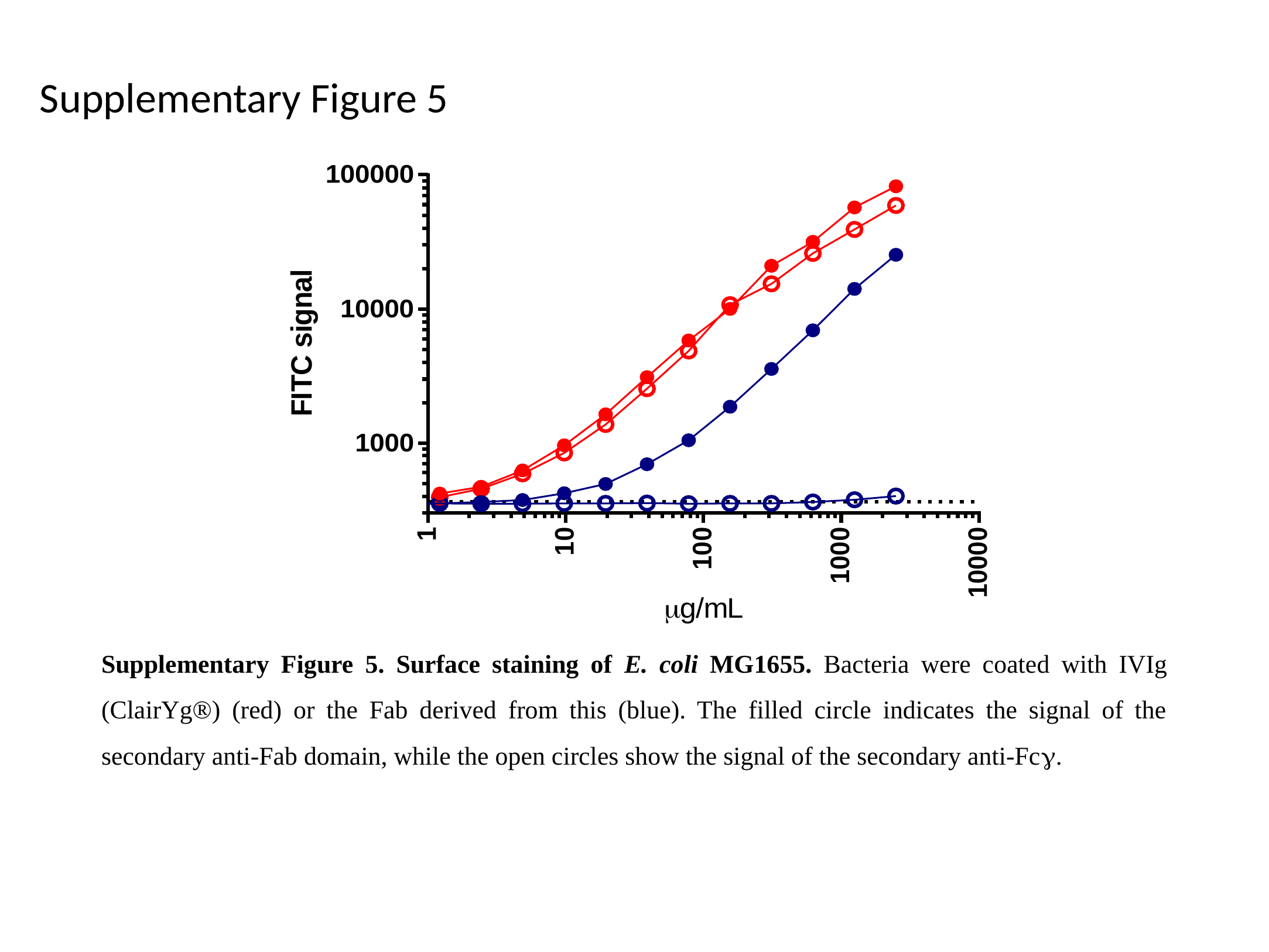

Supplementary Figure 5
Supplementary Figure 5. Surface staining of E. coli MG1655. Bacteria were coated with IVIg (ClairYg®) (red) or the Fab derived from this (blue). The filled circle indicates the signal of the secondary anti-Fab domain, while the open circles show the signal of the secondary anti-Fcg.

## Slide 6
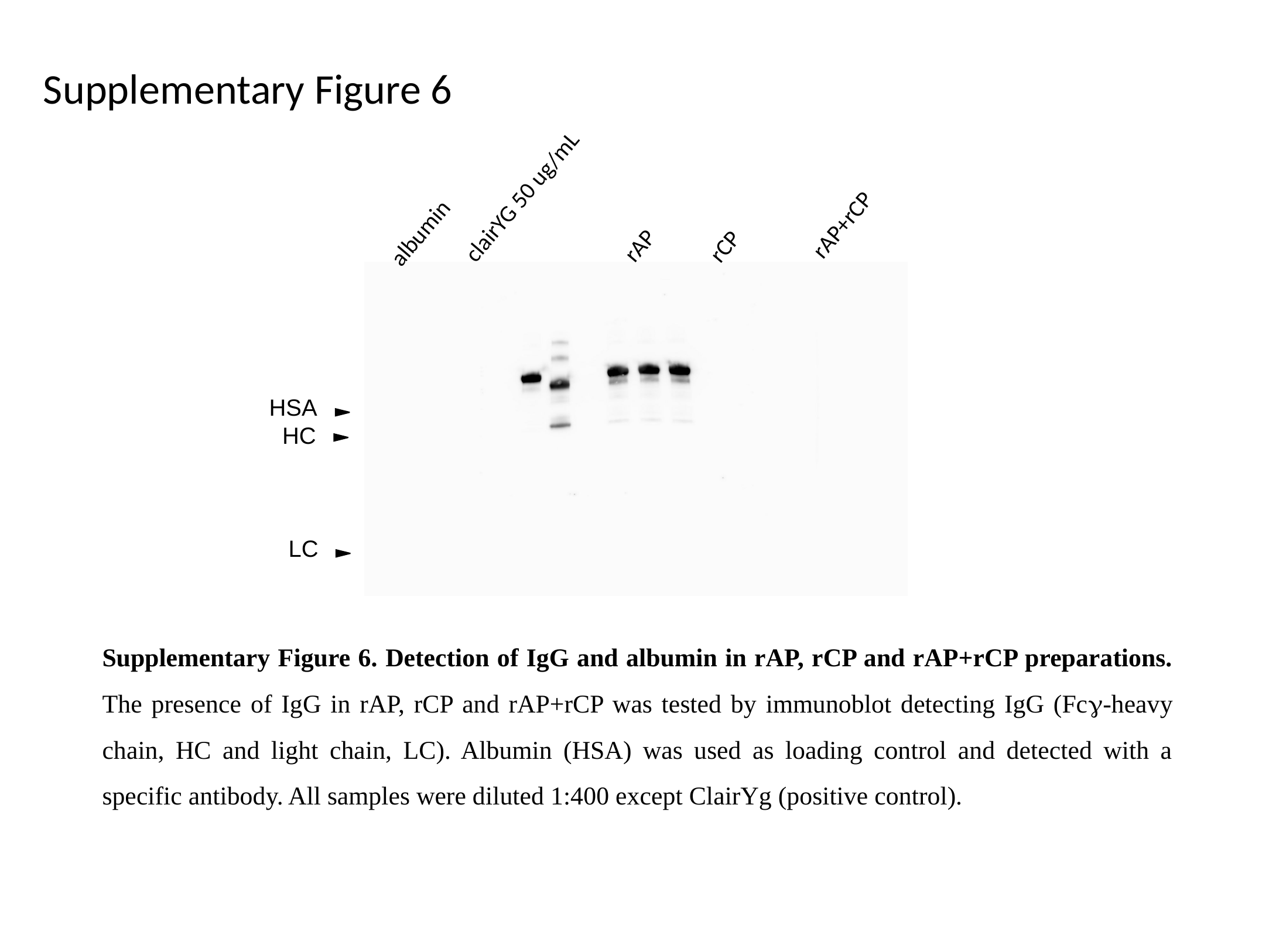

Supplementary Figure 6
rAP+rCP
clairYG 50 ug/mL
rAP
rCP
albumin
HSA
HC
LC
Supplementary Figure 6. Detection of IgG and albumin in rAP, rCP and rAP+rCP preparations. The presence of IgG in rAP, rCP and rAP+rCP was tested by immunoblot detecting IgG (Fcg-heavy chain, HC and light chain, LC). Albumin (HSA) was used as loading control and detected with a specific antibody. All samples were diluted 1:400 except ClairYg (positive control).
